# Habitual dietary intake of IBD patients differs from population controls: a case–control study

**DOI:** 10.1007/s00394-020-02250-z

**Published:** 2020-04-24

**Authors:** Vera Peters, Ettje F. Tigchelaar-Feenstra, Floris Imhann, Jackie A. M. Dekens, Morris A. Swertz, Lude H. Franke, Cisca Wijmenga, Rinse K. Weersma, Behrooz Z. Alizadeh, Gerard Dijkstra, Marjo J. E. Campmans-Kuijpers

**Affiliations:** 1 Department of Gastroenterology and Hepatology, University Medical Centre Groningen and University of Groningen, Hanzeplein 1, P.O. box 30.001, 9700RB Groningen, The Netherlands; 2 Department of Epidemiology, University Medical Centre Groningen and University of Groningen, Groningen, The Netherlands; 3 Department of Genetics, University Medical Centre Groningen and University of Groningen, Groningen, The Netherlands; 4 Top Institute Food and Nutrition, Wageningen, The Netherlands

**Keywords:** Inflammatory bowel disease (IBD), Dietary assessment, Food frequency questionnaire (FFQ), Multinomial logistic regression analysis

## Abstract

**Background:**

Since evidence-based dietary guidelines are lacking for IBD patients, they tend to follow “unguided” dietary habits; potentially leading to nutritional deficiencies and detrimental effects on disease course. Therefore, we compared dietary intake of IBD patients with controls.

**Methods:**

Dietary intake of macronutrients and 25 food groups of 493 patients (207 UC, 286 CD), and 1291 controls was obtained via a food frequency questionnaire.

**Results:**

38.6% of patients in remission had protein intakes below the recommended 0.8 g/kg and 86.7% with active disease below the recommended 1.2 g/kg. Multinomial logistic regression, corrected for age, gender and BMI, showed that (compared to controls) UC patients consumed more meat and spreads, but less alcohol, breads, coffee and dairy; CD patients consumed more non-alcoholic drinks, potatoes, savoury snacks and sugar and sweets but less alcohol, dairy, nuts, pasta and prepared meals. Patients with active disease consumed more meat, soup and sugar and sweets but less alcohol, coffee, dairy, prepared meals and rice; patients in remission consumed more potatoes and spreads but less alcohol, breads, dairy, nuts, pasta and prepared meals.

**Conclusions:**

Patients avoiding potentially favourable foods and gourmandizing potentially unfavourable foods are of concern. Special attention is needed for protein intake in the treatment of these patients.

**Electronic supplementary material:**

The online version of this article (10.1007/s00394-020-02250-z) contains supplementary material, which is available to authorized users.

## Introduction

Inflammatory Bowel Diseases (IBD), comprising ulcerative colitis (UC) and Crohn’s disease (CD), are tedious and incapacitating disorders, impairing quality of life of patients and raising healthcare costs for society [1, 2]. IBD is characterized by mucosal inflammation and ulceration of the gastrointestinal tract. In addition, IBD is known for its erratic course and its varying disease behaviour; periods of severe sickness are alternated with remission [3, 4].

Recent advances in the study of genetics, gut microbiome and environmental factors show that the aetiology of IBD is highly complex and remains to be elucidated. Accumulating evidence points to a dysbiosis of the gut microbiota [5–7] and an aberrant immune response [8] in genetically predisposed individuals [9]; a process probably triggered and maintained by changes in environmental factors, including diet [10, 11]. The Western diet, defined by high intakes of fats and sugars and low intakes of fruits and vegetables [12–17] is proposed as an important risk factor. In addition, high intakes of energy, dairy and cheese [18] are also suggested as potential dietary risk factors. In contrast, fibres [19, 20], fruits [21–23], vegetables [21, 22, 24], fish oil [21, 23, 25], and nuts [21, 23] are considered as potential beneficial factors. Despite comprehensive research on a variety of different dietary elements, it is hard to reach consensus since the majority of studies are bound to recall bias [26] and methodological issues [20, 26, 27]. Therefore, current understanding of dietary effects are too inconsistent to be applicable in clinical practice [17, 28]. Subsequently, the recurrent question of patients: “What should I eat?” remains difficult to answer [29].

Despite the lack of dietary consensus, patients with IBD do not feel intuitively restrained to experiment with their diet in response to symptoms, while they are often already malnourished [30, 31]. Additionally, dietary needs of patients may differ from the general population. We know that protein requirements are different for IBD patients with active disease (1.2–1.5 g/kg) when compared to the general population or IBD patients in remission (0.8–1.0 g/kg) [32–34]. When avoidance of certain food is not supported properly, it may result in nutritional deficiencies; the most common micronutrient deficiencies in IBD due to inadequate dietary intake are iron, calcium, magnesium, vitamin B9, vitamin D and vitamin K [35]. Commonly avoided products by patients include alcohol, fried foods, fruits and nuts [30, 31, 36]. Alcohol and fried foods have been previously suggested as risk factors. However, fruits and nuts were proposed as protective factors [21–23]. To evaluate whether these patients’ experiments deviate from common (allegedly “healthy”) dietary habits, a well-designed comparison between the diet of IBD patients after diagnosis and the diet of the general population is needed.

Here, we aimed to study the patients’ post-diagnosis habitual dietary intake compared to that of the general population in the same geographical area using a Food Frequency Questionnaire (FFQ). This study focusses on differences in dietary intake of nutrients and food groups between disease phenotype and disease activity subgroups compared to population-based controls.

## Materials and methods

### Study setting

This case control study was embedded within the Groningen 1000IBD cohort [37] and the Lifelines DEEP Cohort (LLD) [38]. The 1000IBD cohort is part of the “Parelsnoer” Initiative (PSI) [39], established by the Dutch Federation of University Medical Centre to optimize clinical bio-banking within the eight Dutch university medical centres for research purposes. As part of PSI protocols (described elsewhere [40]), IBD patients are monitored closely and followed-up prospectively using structured questionnaires and standardized approaches.

### Study population

**Fig. 1:**
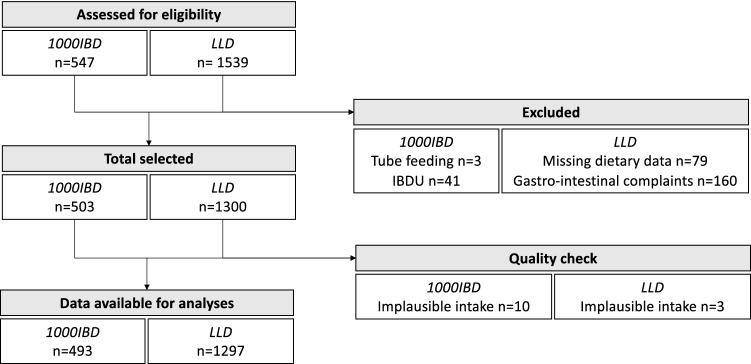
Flowchart inclusion study population

In the context of PSI, dietary data of outpatients of the 1000IBD cohort were obtained via an FFQ [41, 42]. For 547 patients within the 1000IBD cohort dietary data were available and their eligibility was screened for this study, Fig. 1. Participants on tube feeding (*n* = 3) or diagnosed with unclassified IBD (*n* = 41) have been excluded. Next, a quality check was performed; patients (*n* = 10) who had implausible intake regarding to estimated energy intake (< 800 and > 5000 kcal/day [43]) were excluded from analyses. Finally, 493 (61% women; 207 UC, and 286 CD) were included in this study.

In the context of the LLD study, data on participant characteristics and habitual food intake were already collected, Fig. 1. For 1539 LLD participants’ data were available and their eligibility for this study was checked. Participants of the LLD study who had missing dietary data (*n* = 79) or who reported gastro-intestinal complaints (*n* = 160) have been excluded. During the quality check, controls (*n* = 3) who had implausible intake regarding to the estimated energy intake (< 800 and > 5000 kcal/day) were excluded. In the end, 1297 (56% women) healthy population controls were included in this study.

### Dietary assessment

In both cohorts, information about post-diagnosis habitual dietary intake was collected between 2013 and 2016 by the same FFQ which was developed and validated by the nutritional department of Wageningen University using standardized methods [42, 44]. This semi-quantitative FFQ, containing 110 food items divided into 25 food groups, Table S1, assessed food intakes over the previous month as proxy for habitual dietary intake. The NEVO table (Dutch food composition table) [45] was used to calculate individual mean consumption of food items in grams/day and the macronutrient content of the diet.

### Clinical data

Patients’ information was extracted from medical records; the visit closest to the FFQ-date was used. Information was collected on gender, age, height, weight, Body Mass Index (BMI), smoking status, disease phenotype (diagnosis), disease localization, disease duration, and faecal calprotectin levels. Additionally, patient reported outcome measures (PROM) were extracted; Harvey Bradshaw Index (HBI) and Short Clinical Colitis Activity Index (SCCAI).

### Disease activity

Björkesten et al. [46] stated that a score based on HBI and faecal calprotectin is a promising tool for indicating remission in patients with CD. Therefore, disease activity was assessed in all our patients; clinical remission [47–49] for CD patients and was defined as either a faecal calprotectin level of < 200 mg/g or an HBI < 5. This view was extrapolated to UC patients; clinical remission was defined as either a faecal calprotectin level < 200 mg/g or a SCCAI ≤ 2.

### Data analyses

Data were reported as mean ± SD or as *n* (%) where appropriate. Baseline characteristics and dietary differences between disease phenotype (UC vs CD) and disease activity (active vs remission) and controls were analysed using a *t* test or ANOVA and Tukey HSD or Games-Howell post hoc analysis. Categorical variables were tested with a χ^2^-test. False Discovery Rate (FDR) corrected for multiple testing. The Spearman’s Rank Correlation Coefficient was computed to check whether the dietary intake of our IBD population was too heterogenic in terms of disease duration. This was not the case. Additionally, we checked whether gender affected our analyses by stratifying for gender since dietary behaviour is diversiform between women and men [50] and gender has been noted as a major determinant in carbohydrate and lipid metabolism [51–53]. However, only negligible differences were found. Therefore, only overall analyses are presented in this article. Nonetheless, we decided to correct for gender in the multinomial logistic regression analyses. Thus, multinomial logistic regression analysis was performed on food group intake, correcting for age, gender, and BMI. The program IBM SPSS Statistics Version 25 for Mac OS X was used for all analyses.

### Ethical considerations

In this study, data available from the Lifelines DEEP cohort study and the 1000IBD cohort (falling within the PSI) study have been utilized. These studies are performed under ethical approval of the medical ethics committee of the UMCG (respectively, document no. METC UMCG LLDEEP M12.113965 and document no. METC UMCG 1000IBD 2008.338) and according to the Helsinki Declaration of 1975 as revised in 1983 [54]. All patients provided written informed consent to PSI/1000IBD, all controls have provided written informed consent to Lifelines DEEP. This study was carried out with respect to the Research Code of the UMCG.

## Results

### Baseline characterstics

Of all IBD patients in our study, 207 have been previously diagnosed with UC and 286 with CD, Table 1. There were more women among CD patients (67%, *p* = 0.001) than among controls (56%). The age (mean ± SD) of CD patients (40.7 ± 14.4 years, *p* = 0.001) significantly differed from controls (43.7 ± 13.5 years). Compared to controls, UC patients had a higher weight (80.9 ± 16.3 vs 77.7 ± 14.7 kg, *p* = 0.004) and BMI (26.4 ± 5.3 vs 25.2 ± 4.2, *p* = 0.003); whereas CD patients had a lower height (174 ± 9.8 vs 175 ± 9.6 cm, *p* = 0.002) and weight (75.4 ± 15.6 vs 77.7 ± 14.7 kg, *p* = 0.018). Among CD patients were more smokers (31%, *p* < 0.001), whereas among UC patients were less smokers (10%, *p* = 0.003), compared to controls (19%).

**Table 1:** Demographical and clinical characteristics of the study population

			Disease phenotype		Disease activity		Controls
			UC	CD	Active	Remission	
			*n* = 207	*n* = 286	*n* = 241	*n* = 251	*n* = 1291
Gender (female)		*n* (%)	109 (53)	192 (67)*^†^	149 (62)	151 (60)	724 (56)
Age (years)		Mean (SD)	45.6 (13.7)	40.7 (14.4)*^†^	43.2 (15.1)	42.1 (13.7)	43.7 (13.5)
Height (cm)		Mean (SD)	175 (9.9)	174 (9.8)*^†^	175 (10.0)	174 (9.8)*	175 (9.6)
Weight (kg)		Mean (SD)	80.9 (16.3)*	75.4 (15.6)*^†^	77.9 (16.5)	77.5 (15.8)	77.7 (14.7)
BMI		Mean (SD)	26.4 (5.3)*	25.0 (4.7)^†^	25.9 (5.3)	25.6 (4.8)	25.2 (4.2)
Smoking (yes)^∘^		*n* (%)	17 (10)*	63 (31)*^†^	43 (24)	20 (11)	242 (19)
Diagnosis							
	UC	*n* (%)	207 (100)	^−^	94 (39)	113 (45)^‡^	^−^
	CD	*n* (%)	^−^	286 (100)	147 (61)	138 (55)	^−^
Montreal classification							
Age							
	A1	*n* (%)	18 (9)	45 (16)^†^	27 (11)	36 (14)	
	A2	*n* (%)	122 (59)	190 (66)	155 (64)	157 (63)	
	A3	*n* (%)	60 (29)	45 (16)	53 (22)	52 (21)	
Localization							
	L1	*n* (%)	0 (0)	100 (37)^†^	45 (20)	55 (23)	
	L2	*n* (%)	192 (97)	65 (24)	118 (52)	139 (57)	
	L3	*n* (%)	6 (3)	107 (39)	65 (29)	48 (20)	
	L4	*n* (%)	1 (1)	26 (9)^†^	13 (5)	14 (6)	
Behaviour							
	B1	*n* (%)	–	147 (51)	79 (33)	68 (27)	
	B2	*n* (%)	–	98 (34)	48 (20)	50 (20)	
	B3	*n* (%)	–	40 (14)	20 (8)	20 (8)	
	P	*n* (%)	7 (3)	89 (31)^†^	39 (16)	57 (23)	
Extent (UC)							
	E1	*n* (%)	27 (13)	–	11 (5)	16 (6)	
	E2	*n* (%)	64 (31)	–	27 (11)	37 (15)	
	E3	*n* (%)	110 (53)	–	54 (22)	56 (22)	
Severity (UC)							
	S0	*n* (%)	9 (4)	–	5 (2)	4 (2)	
	S1	*n* (%)	62 (30)	–	26 (11)	36 (14)	
	S2	*n* (%)	79 (38)	–	41 (17)	38 (15)	
	S3	*n* (%)	54 (26)	–	21 (9)	33 (13)	
Disease duration (years)		mean (SD)	11.9 (9.3)	12.2 (8.6)	12.2 (9.1)	12.9 (9.2)	^−^
HBI		mean (SD)	^−^	3.49 (3.66)	4.54 (4.07)	1.54 (1.30)^‡^	^−^
SCCAI		mean (SD)	1.56 (1.92)	^−^	2.71 (2.43)	0.64 (0.77)^‡^	^−^
Faecal calprotectin (µg/g)		mean (SD)	491 (1721)	323 (522)	678 (1216)	77 (48)^‡^	^−^

^∘^Deviant number (*n*): smoking: UC = 164, CD = 203, active = 180, remission = 187, controls = 1281, nominal significant difference (*p* < 0.05): *between subgroup vs controls, ^†^between CD vs UC, ^‡^between active vs remission; Montreal classification: A1: ≤ 16 years, A2: 17–40 year, A3: > 40 years. Disease activity: active disease: HBI ≥ 5/SCCAI > 2/faecal calprotectin ≥ 200, disease in remission: HBI < 5/SCCAI ≤ /faecal calprotectin < 200

*L1* terminal ileum, *L2* colon, *L3* ileocolic/backwash ileitis, *L4* isolated upper disease, *B1* non-stricturing, non-penetrating, *B2* stricturing, *B3* penetrating, *P* perianal disease, *E1* ulcerative proctitis, *E2* left-sided, *E3* extensive, *S0* clinical remission, *S1* mild, *S2* moderate, *S3* severe; *HBI* Harvey Bradshaw Index; *SCCAI* short clinical colitis activity index

The mean SCCAI of UC patients was 1.56 ± 1.92 and the mean HBI of CD patients was 3.49 ± 3.66. When we analysed the disease phenotype subgroups reciprocally (UC vs CD), significant differences were observed in gender (109 (53%) vs192 (67%), *p* = 0.001), age (45.6 ± 13.7 vs 40.7 ± 14.4, *p* = 0.001) and Montreal age (A1: 18 (9%) vs 45 (16%), A2: 122 (59%) vs 190 (66%), A3: 60 (29%) vs 45 (16%), *p* = 0.001) height (175 ± 9.9 vs 174 ± 9.8, *p* = 0.041), weight (80.9 ± 16.3 vs 75.4 ± 15.6, *p* < 0.001), BMI (26.4 ± 5.3 vs 25.0 ± 4.7, *p* = 0.004), smoking status (17 (10%) vs 63 (31%), *p* < 0.001) and naturally in disease localization (ileal: 0 (0%) vs 100 (37%), colonic: 192 (97%) vs 65 (24%), ileocolonic/backwash ileitis: 6 (3%) vs 107 (39%), *p* < 0.001 and isolated upper disease: 1 (1%) vs 26 (9%), *p* < 0.001) and perianal disease (97 (3%) vs 89 (31%), *p* < 0.001) (Table 1).

When we compared disease activity subgroups with controls, only height (174 ± 9.8 vs 175 ± 9.6 cm, *p* = 0.041) in patients in remission was significantly different.

Comparing disease activity subgroups (active vs remission), naturally patients with active disease had higher mean SCCAI (2.71 ± 2.43, *p* < 0.001), HBI (4.54 ± 4.07, *p* < 0.001) and calprotectin levels (678 ± 1216, *p* < 0.001) from patients in clinical remission; SCCAI (0.64 ± 0.77), HBI (1.54 ± 1.30), calprotectin (76.7 ± 48.0).

### Macronutrients

Macronutrient intake of disease phenotype subgroups, Table 2 differed significantly in the intake of controls; UC patients had a lower intake of total protein (0.91 ± 0.30 vs 0.97 ± 0.28 g/kg, *p* = 0.017), animal protein (0.52 ± 0.19 vs 0.57 ± 0.20 g/kg, *p* = 0.005), carbohydrates (45.3 ± 6.0 vs 46.6 ± 5.9 En%, *p* = 0.009) and alcohol (4.1 ± 5.9 vs 8.2 ± 9.2 g, *p* < 0.001) but had a higher intake of fat (36.2 ± 5.5 vs 35.1 ± 5.0 En%, *p* = 0.026). CD patients also had a lower intake of total protein (0.90 ± 0.31 vs 0.97 ± 0.28 g/kg, *p* = 0.001), animal protein (0.52 ± 0.21 vs 0.57 ± 0.20 g/kg, *p* < 0.001) and alcohol (4.0 ± 6.9 vs 8.2 ± 9.2 g, *p* < 0.001), but not of carbohydrates. However, CD patients additionally showed a lower intake of plant protein (28.2 ± 10.9 vs 30.5 ± 9.9 g, *p* = 0.002) and showed no higher fat intake compared to controls.

**Table 2:** Macronutrient intake of patients and controls

	Disease phenotype		Disease activity		Controls
	UC	CD	Active	Remission	
	*n* = 207	*n* = 286	*n* = 241	*n* = 251	*n* = 1291
Kilocalories	1997 (585)	1897 (639)	1925 (656)	1954 (582)	1959 (569)
BMR%	124 (35.2)	122 (41.3)	122 (39.9)	124 (38.1)	121 (33.5)
Total protein	71.9 (19.7)	66.0 (20.0)*^†^	67.9 (21.1)*	69.0 (19.1)*	73.8 (20.2)
g/kg	0.91 (0.30)*	0.90 (0.31)*	0.89 (0.30)*	0.92 (0.31)*	0.97 (0.28)
Plant protein	30.7 (10.5)	28.2 (10.9)*^†^	28.6 (11.0)*	29.9 (10.7)	30.5 (9.9)
g/kg	0.39 (0.16)	0.39 (0.16)	0.38 (0.15)*	0.40 (0.17)	0.40 (0.14)
Animal protein	41.3 (13.8)	37.8 (13.4)*^†^	39.3 (14.4)*	39.2 (13.0)*	43.3 (14.6)
g/kg	0.52 (0.19)*	0.52 (0.21)*	0.52 (0.21)*	0.52 (0.20)*	0.57 (0.20)
Fat	80.9 (28.4)	75.1 (30.6)	77.2 (30.8)	78.0 (28.9)	77.2 (27.3)
En%	36.2 (5.5)*	35.3 (5.7)	35.8 (5.1)	35.6 (6.1)	35.1 (5.0)
Carbohydrates	226 (75.3)	221 (82.3)	222 (81.5)	225 (77.7)	228 (70.5)
En%	45.3 (6.0)*	46.6 (6.7)^†^	46.1 (5.8)	45.9 (7.0)	46.6 (5.9)
Alcohol^1^	4.1 (5.9)*	4.0 (6.9)*	3.7 (6.6)*	4.4 (6.4)*	8.2 (9.2)
En%^1^	1.5 (2.3)*	1.5 (2.5)*	1.4 (2.3)*	1.7 (2.6)*	3.0 (3.2)

Italic represents differences that are only nominal significant and not statistical significant

Observed variables are shown as mean (SD) in grams/day, ^1^statistics were performed on √-transformed variables, where appropriate ANOVA or Welch and post hoc Tukey HSD tests, or Games–Howell tests are interpreted; BMR% = Total kilocalorie intake as percentage of Basal Metabolic Rate (Calculated with Harris Benedict Equation); women: BMR = 655.0955 + (9.5634 × Weight) + (1.8496 × Length) − (4.6756 × Age); men: BMR = 66.4730 + (13.7516 × Weight) + (5.0033 × Length) − (6.7550*Age); En% = Macronutrient as percentage of total kilocalorie intake; nominal (*p* < 0.05) and statistical (FDR corrected; false discovery rate = 0.05) significant difference: *between subgroup vs controls, ^†^between CD vs UC, ^‡^between active vs remission

UC patients differed from CD patients in the intake of total protein (71.9 ± 19.7 vs 66.0 ± 20.0 g, *p* = 0.004), plant protein (30.7 ± 10.5 vs 28.2 ± 10.9 g, *p* = 0.019), animal protein (41.3 ± 13.8 vs 37.8 ± 13.4 g, *p* = 0.023) and carbohydrates (45.3 ± 6.0 vs 46.6 ± 6.7 En%, *p* = 0.041).

**Fig. 2:**
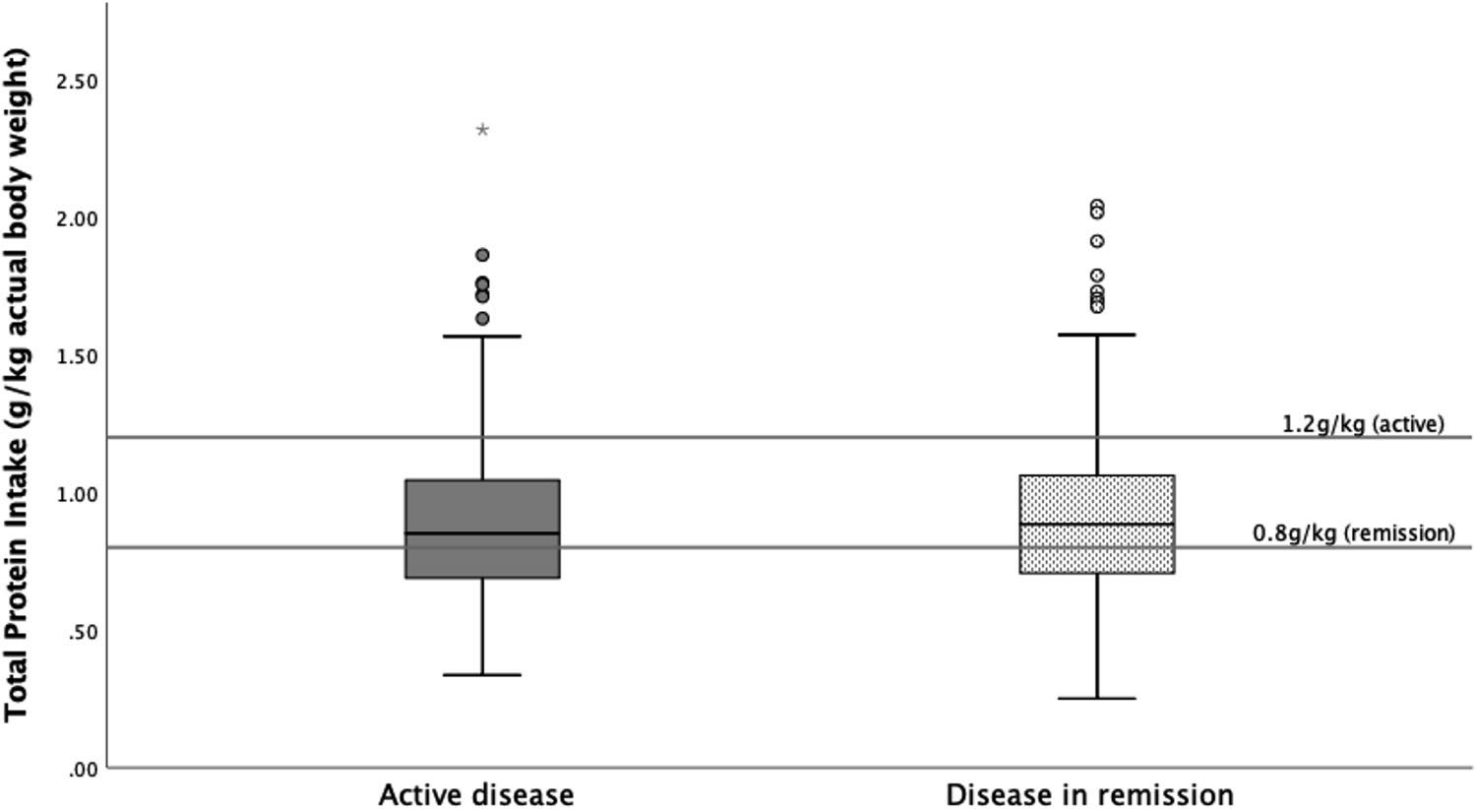
Total protein intake of patients and recommended protein requirements

Macronutrient intake of disease activity subgroups differed significantly to intake of controls; patients with active disease consumed less total protein (0.89 ± 0.30 g/kg vs 0.97 ± 0.28 g/kg, *p* < 0.001), plant protein (0.38 ± 0.15 vs 0.40 ± 0.14 g/kg, *p* = 0.037), animal protein (0.52 ± 0.21 vs 0.57 ± 0.20 g/kg, *p* = 0.001) and alcohol (3.7 ± 6.6 vs 8.2 ± 9.2 g, *p* < 0.001). Of the patients with active disease, 86.7% had a protein intake < 1.2 g/kg (Fig. 2, Table 2, Table S2**)**. Patients in remission had lower intakes of total protein (0.92 ± 0.31 vs 0.97 ± 0.28 g/kg, *p* = 0.024), animal protein (0.52 ± 0.20 vs 0.57 ± 0.20 g/kg, *p* = 0.001) and alcohol (4.4 ± 6.4 vs 8.2 ± 9.2 g, *p* < 0.001). Of the patients in remission, 38.6% had a protein intake < 0.8 g/kg (Fig. 2, Tables 2 and 3).

No differences were seen in macronutrient intake when comparing disease activity subgroups; patients in remission vs patients with active disease.

### Food groups—ANOVA

Comparing disease phenotype subgroups to controls, statistically significant differences were observed in 9 food groups consumed by UC patients and in 15 food groups consumed by CD patients, Table 3. Compared to controls, **UC patients** had lower intakes of alcohol (57.0 ± 94.5 vs 116 ± 159, *p* < 0.001), coffee (284 ± 230 vs 382 ± 275, *p* < 0.001), dairy (250 ± 171 vs 285 ± 192, *p* = 0.014), nuts (11.8 ± 18.0 vs 14.3 ± 15.7, *p* = 0.004), pasta (22.5 ± 24.1 vs 28.1 ± 24.0, *p* < 0.001), prepared meals (46.9 ± 65.8 vs 59.4 ± 57.8, *p* < 0.001) and rice (22.5 ± 29.2 vs 27.7 ± 28.8, *p* < 0.001); and higher intakes of meat (90.9 ± 46.1 vs 82.0 ± 42.3, *p* = 0.048) and potatoes (90.4 ± 60.9 vs76.1 ± 50.8, *p* = 0.016). Compared to controls, **CD patients** consumed less alcohol (54.6 ± 105 vs 116 ± 159, *p* < 0.001), breads (126 ± 73.9 vs 135 ± 63.2, *p* = 0.014), cereals (4.74 ± 11.3 vs 7.26 ± 13.3, *p* < 0.001), cheese (26.2 ± 27.1 vs 30.2 ± 26.8, *p* = 0.006)), coffee (304 ± 282 vs 382 ± 275, *p* < 0.001), dairy (214 ± 195 vs 285 ± 192, p < 0.001), nuts (10.2 ± 16.9 vs 14.3 ± 15.7, *p* < 0.001), pasta (18.9 ± 20.5 vs 28.1 ± 24.0, *p* < 0.001), pastry (29.0 ± 30.4 vs 31.9 ± 23.3, *p* < 0.001), prepared meals (43.2 ± 47.8 vs 59.4 ± 57.8, *p* < 0.001), rice (22.1 ± 29.6 vs 27.7 ± 28.8, *p* < 0.001), sauces (14.8 ± 16.8 vs 16.7 ± 14.6, *p* = 0.015) and vegetables (102 ± 73.1 vs 108 ± 63.6, *p* = 0.049) but more non-alcoholic drinks (225 ± 269 vs 155 ± 196, *p* < 0.001) and sugar and sweets (38.0 ± 33.8 vs 32.4 ± 26.8, *p* = 0.032).

**Table 3:** Univariate comparison of food group intake between patients and controls

Food groups^1^	Disease phenotype		Disease activity		Controls
	UC	CD	Active	Remission	
	*n* = 207	*n* = 286	*n* = 241	*n* = 251	*n* = 1291
Alcohol	57.0 (94.5)*	54.6 (105)*	51.9 (102)*	59.2 (100)*	116 (159)
Breads	136 (67.3)	126 (73.9)*	127 (74.5)	133 (68.3)	135 (63.2)
Cereals	6.78 (14.4)	4.74 (11.3)*	5.20 (13.0)*	5.95 (12.5)	7.26 (13.3)
Cheese	28.1 (24.0)	26.2 (27.1)*	27.2 (28.8)*	26.5 (22.2)	30.2 (26.8)
Coffee	284 (230)*	304 (282)*	303 (269)*	290 (254)*	382 (275)
Dairy	250 (171)*	214 (195)*^†^	229 (185)*	227 (186)*	285 (192)
Eggs	14.2 (15.2)	13.9 (13.5)	15.1 (15.6)	13.0 (12.7)	14.4 (14.5)
Fish	14.9 (15.7)	14.2 (16.1)	15.0 (17.5)	14.1 (14.3)	15.1 (16.0)
Fruits	219 (179)	229 (195)	229 (186)	220 (191)	223 (159)
Legumes	11.7 (18.2)	*9.24 (22.0)*^†^	10.6 (24.5)	9.90 (15.8)	9.41 (15.8)
Meat	90.9 (46.1)*	82.2 (42.4)	85.1 (46.4)	86.8 (41.7)	82.0 (42.3)
Non-alcoholic drinks	166 (244)	225 (269)*^†^	195 (258)	204 (263)*	155 (196)
Nuts	11.8 (18.0)*	10.2 (16.9)*	8.80 (11.7)*	12.9 (21.3)*^‡^	14.3 (15.7)
Pasta	22.5 (24.1)*	18.9 (20.5)*	18.5 (18.0)*	22.3 (25.4)*	28.1 (24.0)
Pastry	33.3 (25.0)	29.0 (30.4)*	31.8 (32.5)	29.8 (23.7)	31.9 (23.3)
Potatoes	90.4 (60.9)*	84.9 (68.3)	92.6 (71.9)*	82.1 (58.1)	76.1 (50.8)
Prepared meals	46.9 (65.8)*	43.2 (47.8)*	43.8 (48.4)*	45.7 (62.7)*	59.4 (57.8)
Rice	22.5 (29.2)*	22.1 (29.6)*	22.1 (30.0)*	22.6 (29.0)*	27.7 (28.8)
Sauces	15.6 (17.1)	14.8 (16.8)*	14.9 (17.8)	15.3 (16.1)*	16.7 (14.6)
Savoury snacks	17.5 (15.8)	19.1 (22.3)	18.8 (21.6)	18.1 (18.0)	17.7 (16.4)
Soup	52.9 (64.2)	42.8 (49.1)	45.1 (57.8)	49.0 (54.5)	45.9 (54.8)
Spreads	24.3 (18.6)	22.2 (19.6)	23.5 (19.7)	22.7 (18.7)	21.7 (15.9)
Sugar and sweets	36.2 (28.2)	38.0 (33.8)*	36.2 (31.0)	38.4 (32.1)*	32.4 (26.8)
Tea	279 (257)	268 (267)	265 (253)	279 (273)	243 (242)
Vegetables	108 (65.5)	102 (73.1)*	104 (71.8)	105 (68.5)	108 (63.6)

Italic represents differences that are only nominal significant and not statistical significant

Observed variables are shown as mean (SD) in grams/day, ^1^statistics were performed on √-transformed variables; where appropriate ANOVA or Welch and post hoc Tukey HSD tests, or Games–Howell tests are interpreted; nominal (*p* < 0.05) and statistical (FDR corrected; false discovery rate = 0.05) significant difference: *between subgroup vs controls, ^†^between CD vs UC, ^‡^between active vs remission

Comparing disease phenotype groups reciprocally (**UC vs CD**), UC patients consumed more dairy (250 ± 171 vs 214 ± 195, *p* = 0.018) and legumes (11.7 ± 18.2 vs 9.24 ± 22.0, *p* = 0.016); but less non-alcoholic drinks (166 ± 244 vs 225 ± 269, *p* = 0.003).

Disease activity subgroups were compared to controls as well; 10 differences were found comparing active disease and remission to controls. Compared to controls, **patients with active disease** consumed less alcohol (51.9 ± 102 vs 116 ± 159, *p* < 0.001), cereals (5.20 ± 13.0 vs 7.26 ± 13.3, *p* = 0.002), cheese (27.2 ± 28.8 vs 30.2 ± 26.8, *p* = 0.030), coffee (303 ± 269 vs 382 ± 275, *p* < 0.001), dairy (229 ± 185 vs 285 ± 192, *p* < 0.001), nuts (8.80 ± 11.7 vs 14.3 ± 15.7, *p* < 0.001), pasta (18.5 ± 18.0 vs 28.1 ± 24.0, *p* < 0.001), prepared meals (43.8 ± 48.4 vs 59.4 ± 57.8, *p* < 0.001) and rice (22.1 ± 30.0 vs 27.7 ± 28.8, *p* < 0.001) but more potatoes (92.6 ± 71.9 vs 76.1 ± 50.8, *p* = 0.016). **Patients in remission** consumed less alcohol (59.2 ± 100 vs 116 ± 159, *p* < 0.001), coffee (290 ± 254 vs 382 ± 275, *p* < 0.001), dairy (227 ± 186 vs 285 ± 192, *p* < 0.001), nuts (12.9 ± 21.3 vs 14.3 ± 15.7, *p* = 0.012), pasta (22.3 ± 25.4 vs 28.1 ± 24.0, *p* < 0.001), prepared meals (45.7 ± 62.7 vs 59.4 ± 57.8, *p* < 0.001), rice (22.6 ± 29.0 vs 27.7 ± 28.8, *p* < 0.001) and sauces (15.3 ± 16.1 vs 16.7 ± 14.6, *p* = 0.047) but more non-alcoholic drinks (204 ± 263 vs 155 ± 196, *p* = 0.041) and sugar and sweets (38.4 ± 32.1 vs 32.4 ± 26.8, *p* = 0.011).

Comparing disease activity groups reciprocally (**active vs remission**); patients with active disease consumed less nuts (8.80 ± 11.7 vs 12.9 ± 21.3, *p* = 0.010) than those in remission.

### Food groups—multinomial logistic regression

Results of the multinomial logistic regression analyses, correcting for age, gender and BMI, are shown in Table S3. Compared to controls, it was more likely that UC patients had a higher intake of meat and spreads, but a lower intake of alcohol, breads, coffee and dairy; CD patient consumed more non-alcoholic drinks, potatoes, savoury snacks and sugar and sweets but less alcohol, dairy, nuts, pasta and prepared meals. Moreover, compared to controls, patients with active disease tend to eat/drink more meat, soup and sugar and sweets but less alcohol, coffee, dairy, prepared meals and rice; patients in remission took in more potatoes and spreads but less alcohol, breads, dairy, nuts, pasta and prepared meals.

## Discussion

To summarize our finding: patients consumed less protein than controls; 38.6% of patients in remission had a protein intake < 0.8 g/kg and 86.7% with active disease had a protein intake < 1.2 g/kg. Furthermore, patients consumed less potentially unfavourable food groups such as alcohol, pasta and prepared meals. But they consumed more of potentially unfavourable food groups such as meat, non-alcoholic drinks, and sugar & sweets. Moreover, a higher intake of savoury snacks and spreads was reported in patients. Additionally, patients avoided potential favourable foods such as breads, coffee, dairy, nuts and rice. However, they consumed more of the potential favourable foods such as potatoes and soup.

The guidelines from the European Society for Clinical Nutrition and Metabolism (ESPEN) recommend adult IBD patients with active disease to increase their protein requirement to 1.2–1.5 g/kg body weight/day [32], whereas in the general population only 0.8 g/kg body weight/day is recommended [33, 34]. Despite these increased recommendations, we observed that our IBD patients had a lower protein intake than controls; 86.7% of the patients with active disease did not meet the increased recommended protein intake of 1.2 g/kg/day. This higher intake is recommended in these patients due to protein losses caused by e.g. inflammation in the gastrointestinal tract [33]. Too low protein intake might lead to muscle mass loss. Recently, it was demonstrated that appendicular skeletal muscle index, an indicator of muscle mass, significantly decreases in patients within two year after being diagnosed with IBD [55]. Besides, these patients tend to consume less animal protein which is of higher nutritional value, since animal protein, in contrast to plant protein, contains all essential amino-acids. On the other hand, high protein intake could impact the gut microbiota composition and thereby inducing some typical feature of IBD-associated dysbiosis [34]. In addition to quantity, it might be that the source of proteins (animal not plant) may modulate the risk [34]. Among animal protein sources, high intakes of meat or fish but not of eggs or dairy products has been related to the risk on IBD [56]. Therefore, the source of protein should be taken into consideration when advising patients with IBD to increase their protein intake in a allegedly healthy manner (i.e. complementation of plant protein sources [57]).

Since evidence-based dietary guidelines are lacking for patients with IBD, proper research into dietary effects on IBD and its course is urgently needed. We studied patients’ habitual dietary intake in comparison to controls. In the light of current available evidence, we will discuss the intake of food groups as potentially “unfavourable” or “favourable”, since the intake of unguided diets may lead to potential unwanted effects on disease course.

We reported that patients had lower intakes of potential unfavourable foods like alcohol, pasta and prepared meals. Avoidance of alcohol is one of these sensible alterations, consumption of alcohol increases the risk on relapse by 2.7 times [58]. Thus, this alteration may help patients to stay in remission. Furthermore, prepared meals are demonstrated to lead to a 2.9-fold increased risk of UC, and 2.3-fold of CD [59]. On the other hand, IBD patients have higher intakes of certain potential unfavourable foods. Our patients consumed more meat, non-alcoholic drinks and sugar and sweets. Although meat, especially red and processed meat, was identified as potential risk for IBD [56, 58, 60–64], our UC patients and those with active disease had a higher meat intake which can potentially lead to aggravation of symptoms [31, 36, 65]. The higher consumption of non-alcoholic beverages might be compensatory to a lower intake of alcoholic beverages. This is of concern, as some non-alcoholic beverages have been reported as risk factor for IBD; for example, cola, a popular non-alcoholic drink, was reported to increase the risk for UC 1.6 times and that for CD 2.2 times [66]. Several studies [21, 22, 67] identified sugar as a risk factor for IBD. Unfortunately, our CD patients and patients with active disease consumed more sugar and sweets when compared to controls. Perhaps, this might be explained by the invisibility of sugars. They may consume more sugar than they realize when they are using (alternative) products. Furthermore, there is not enough evidence available to classify savoury snacks and spreads as potential (un)favourable food groups [68]. Savoury snacks are reported to be consumed more by CD patients than controls. Spreads are consumed frequently by CD patients [68], and also by our UC patients and patients in remission.

Additionally, patients avoided potentially favourable foods too; patients consumed less breads, coffee and dairy compared to controls. Moreover, less nuts were consumed by CD patients and patients who are in remission. Besides, less rice was eaten by patients with active disease. Breads and cereals are major sources of fibre and are identified as protective to IBD [22]. Only a few studies reported patients having experienced complaints due to intake of bread and cereals [30, 65]. Nevertheless, patients avoided these food groups. This might be due to a hype among IBD patients of adapting a gluten-free diet [69]. Besides, yeast, commonly present in bread, is reported to be harmful for IBD patients [70]. Several studies report that patients avoid coffee due to aggravated symptoms [30, 31, 36]. Though, coffee is suggested to be beneficial in IBD in a few studies [67, 71]. However, the exact mechanism of coffee is unclear [72]. In our study we found a lower coffee consumption in UC patients and patients with active disease. Dairy products are reported to be commonly avoided by patients as well [73, 74]. Disease relapse was not associated with dairy intake [75] and as an animal protein source, dairy was not associated with the risk on IBD [56]. Recently, yoghurt, buttermilk and fermented milk have demonstrated an anti-inflammatory effect in vitro [76]. Avoidance of dairy leads to increased risk of low calcium levels and osteoporosis [77], as commonly seen in 20–50% of the IBD patients already [70]. Nuts are a natural source of sulphur which is potentially causing greater risk on relapses in UC patients [19, 58]. Nevertheless, nuts are suggested to be protective in the development of CD [21, 23]. Rice is reported to improve symptoms [31], nonetheless, patients with active disease had a lower intake. Soup on the other hand, is likely consumed more by our patients with active disease as it is reported to be potentially beneficial [31]. Additionally, more potatoes are consumed by CD patients and patients in remission. Potatoes contain glycoalkaloid that aggravated intestinal inflammation in predisposed mice [78]. On the other hand, resistant starch in potatoes might be beneficial in IBD [79]. Superiority of pasta, rice and potatoes to each other in IBD patients cannot be deducted from literature [61]. Pasta was not directly associated with an increased risk on developing UC. However, Maconi et al. [61] reported an positive association between adapting to a “refined” dietary pattern (including pasta, sweets, red and processed meat, butter and margarine) and the risk on developing IBD. In our study, patients often consumed less rice and pasta, but more potatoes.

The patients in our study population seemed to follow “unguided” dietary habits (meaning without guidance of a physician or dietician) based on personal feelings and experiences considering diet and symptoms. Nevertheless, it is possible that these dietary habits were guided, since dietary guidance was not registered during the study period. Moreover, our patient population does include patients with different Montreal classifications, including penetrating/stricturing disease. Although evidence is lacking, clinicians and patients do widely accept that a low-residue diet may be beneficial for IBD patients when having a severe flare or stricturing CD and obstructive symptoms [30, 80]. Hence, this may have influenced habitual dietary intake of patients in our study population. Moreover, patients were classified as active or in remission based on either HBI/SCCAI or faecal calprotectin levels. Hence, the active group had a mean HBI < 5 (generally regarded as clinical remission) but on the other hand a mean faecal calprotectin of > 200 g/mg (cut-off clinical remission). The fact that patients did not have complaints (reflected in the HBI/SCCAI score), may have influenced their eating habits as well. Intakes of IBD patients were compared to intakes of controls. Our control population consists of Lifelines DEEP [38] participants, a subgroup of the Lifelines cohort study. The intake of the complete cohort is described elsewhere and can be considered as representative for the intake in the Netherlands.[81]

We acknowledge the limitations appurtenant to an FFQ, moreover this FFQ did not obtain direct data on fat type, fibre, salt intake and micronutrient intake (e.g. calcium). Furthermore, since IBD patients tend to eat small quantities of food each time, an FFQ might underestimate their true dietary intake. Dietary intake was only assessed once in this study, ignoring seasonal differences in habitual intake, nevertheless FFQs are an appropriate method to assess long-term dietary intake [82]. For future use we recently developed an IBD specific FFQ [83]. Besides, dietary guidance was not registered, therefore the reasoning of patients behind why they adapt to a certain diet is lacking. Strengths of this study are the inclusion of a large population-based sample set embedded within two prospective cohorts, and the use of a validated FFQ to assess dietary intake, while statistical analyses controlled for potential confounding factors like age, gender and BMI. Furthermore, we assessed food intakes of UC and CD patients separately and could distinguish between patients with active disease or in remission.

## Conclusions

In conclusion, our study confirms that there are several relevant differences in habitual dietary intake of patients compared to controls; patients avoid potentially favourable food groups such as breads, coffee, dairy, nuts and rice, and gourmandize potentially unfavourable food groups like meat, non-alcoholic drinks and sugar and sweets. This might attribute to potentially unintended effects on disease course and its complications. To be able to propose better dietary guidelines for IBD patients, more research into dietary effects on IBD disease course is urgently needed. A major concern is the lower protein intake; further attention to protein intake is needed in future studies and in the treatment of these patients, especially in those with active disease when protein needs are increased.

## Electronic supplementary material

Below is the link to the electronic supplementary material.

Supplementary file1 Table S1. Categorization of food items into food groups (PDF 47 kb)

Supplementary file2 Table S2. Total protein intake of patients and controls (PDF 31 kb)

Supplementary file3 Table S3. Multivariate analysis of food group intake in patients and controls (PDF 42 kb)
