# Supplementary material for: Habitual dietary intake of IBD patients differs from population controls: a case–control study
Source: Eur J Nutr. 2020 Apr 24;60(1):345–56. doi: 10.1007/s00394-020-02250-z (PMC7867519; doi:10.1007/s00394-020-02250-z)
Supplement: Supplementary file 1 — Supplementary file1 Table S1. Categorization of food items into food groups (PDF 47 kb) [file 394_2020_2250_MOESM1_ESM.pdf]

Table S1. Categorization of food items into food groups

English item name	Dutch item name	Food group
Beer	Bier	Alcohol
Red wine	Rode wijn	Alcohol
White wine	Witte wijn	Alcohol
Fortified wines: Sherry / port / vermouth / madeira	Sherry/port/vermouth/madeira	Alcohol
Spirit drinks	Gedestilleerde dranken	Alcohol
Other types of alcoholic drinks	Overige soorten alcoholische dranken	Alcohol
Plain rusks / crisp bread / crackers	Beschuit/knäckebröd/crackers	Breads
Croissants / other breads	Croissants/andere broodjes	Breads
Slices of bread / sandwich bread	Sneden brood	Breads
Granola / muesli / cereals	Muesli/cruesli/granen voor pappen	Cereals
20+/30+ cheese/cheese spreads	20+/30+ kaas/smeerkaas	Cheese
40+ cheese/cheese spreads	40+ kaas/smeerkaas	Cheese
48+ full-fat cheese/cheese spreads	48+ volvette kaas/smeerkaas	Cheese
Cream cheese / foreign cheeses	Roomkaas/buitenlandse kaas	Cheese
Cheese added to dinner	Kaas bij warme maaltijd	Cheese
Cheese in-between meals	Kaas tussendoor	Cheese
Coffee	Koffie	Coffee
Ready-made breakfast drinks	Kant en klaar drinkontbijt	Dairy
Whole milk	Volle melk	Dairy
Reduced-fat / semi-skimmed milk	Halfvolle melk	Dairy
Lowe-fat / skimmed milk	Magere melk	Dairy
Buttermilk	Karnemelk	Dairy
Chocolatemilk	Chocolademelk	Dairy
Drinking yoghurt / milk drink with flavor and sugar	Drinkyoghurt/zuiveldrank met smaakje en suiker	Dairy
Other types of dairy drinks	Overige soorten zuiveldrank	Dairy
Whole pudding/custard	Volle vla	Dairy
Whole natural yoghurt	Volle naturel yoghurt	Dairy
Low-fat / skimmed natural yoghurt	Magere naturel yoghurt	Dairy
Low-fat / skimmed (fruit) yoghurt	Magere (vruchten)yoghurt	Dairy
Quark / curd / fruitcurd	Kwark/vruchtenkwark	Dairy
Ready-made pap / porridge	Kant en klaar pap	Dairy
Other types of pudding / custard / yoghurt / curd / quark products	Overige soorten vla/yoghurt/kwark	Dairy
Ice cream / milk-based ice	Roomijs/ijs op melkbasis	Dairy
Whipped cream	Slagroom	Dairy
Halvamel	Halvamel	Dairy
Coffee creamer	Koffiecreamer	Dairy
Whole coffee milk	Volle koffiemelk	Dairy
Ordinary milk (for coffee)	Gewone melk (koffie)	Dairy
Other types of milk / dairy	Overige soorten melk (koffie)	Dairy
Boiled eggs	Gekookt ei	Eggs
Fried eggs	Gebakken ei	Eggs
Salty herring	Zoute haring	Fish
Fried haddock fillet / fried cod bits	Lekkerbekje/kibbeling	Fish
Low-fat / whitefish	Magere vis	Fish
Fatty fish	Vette vis	Fish
Other types of fish	Overige soorten vis	Fish
Self prepared fish with fat	Vis zelf bereid met vet	Fish
Fresh fruit	Vers fruit	Fruits
Apple sauce	Appelmoes	Fruits
Fruit juice	Vruchtensap/-drank	Fruits
Legumes	Peulvruchten	Legumes
Sausage / bacon (cold cuts)	Worst/speksoorten (brood)	Meat
Other types of cold cut meat and sausages	Overige soorten vleeswaren (brood)	Meat
Mince meat	Gehakt	Meat
Smoked sausage	Rookworst	Meat
Steak / roast beef / ox meat	Biefstuk/rosbief/runderlap etc.	Meat
Meat olives / braised meat	Blinde vink/doorregen runderlap etc.	Meat
Pork steak / fried rice meat / schnitzel	Hamlap/nasivlees/schnitzel etc.	Meat
Pork fillet / pork chops	Varkensfiletlap/karbonade etc.	Meat
Pork belly / bacon	Slavink/speklap/spekjes etc.	Meat
Chicken	Kip	Meat
Other types of meats or poultry	Overige soorten vlees of gevogelte	Meat
Gravy	Jus	Meat
Cold meats and sausages (cold cuts) in-between meals	Worst/vleeswaren tussendoor	Meat
Soda / lemonade with sugar	Frisdranken/limonade met suiker	Nonalcoholic drinks
Diet soda / lemonade without sugar	Light frisdranken/limonade zonder suiker	Nonalcoholic drinks
Non-alcoholic beer	Alcoholvrij bier	Nonalcoholic drinks
Peanutbutter	Pindakaas	Nuts
Nuts added to dinner	Pinda's/noten bij warme maaltijd	Nuts
Nuts in-between meals	Pinda's/noten tussendoor	Nuts
Pasta	Pasta	Pasta
Small cookies / biscuits	Kleine koekjes/biscuitjes	Pastry
Cake / large cookies	Cake/grote koeken	Pastry
Pastry / pie	Gebak/taart	Pastry
Gingerbread / cake bars / food biscuits	Ontbijtkoek/koekrepen/voedingsbiscuits	Pastry
Cooked potatoes / mashed potatoes	Gekookte aardappelen/puree	Potatoes
French fries / baked potatoes	Frites/gebakken aardappelen	Potatoes
Chinese / Indean ready-made meals	Chinees/indische k&k	Prepared meals
Fast food	Fast-food maaltijden	Prepared meals

Other types of ready-made meals	Overige soorten k&k	Prepared meals
Pizza	Pizza	Prepared meals
Rice	Rijst	Rice
Warm sauces	Warme sauzen	Sauces
Mayonnaise	Mayonnaise	Sauces
Halvanaise / mayonnaise / non-red sauces/condiments	Halvanaise/fritessaus/niet-rode sauzen	Sauces
Dressing / salad dressing	Dressing/slasaus	Sauces
Clear dressing without oil	Heldere dressing zonder olie	Sauces
Mayonnaise added to snacks	Mayonnaise (snacks)	Sauces
Halvanaise / mayonnaise / non-red sauces/condiments added to snacks	Halvanaise/fritessaus/niet-rode sauzen (snacks)	Sauces
Sandwich spreads / salads (on bread)	Sandwich spread/salade (brood)	savory snacks
Warm savory snacks	Warme hartige snacks	savory snacks
Potato crisps / salty snacks / pretzels	Chips/zoutjes	savory snacks
Sandwich spreads / salads on French bread / toast	Salades op stokbrood/toast	savory snacks
Soup with legumes	Soep met peulvruchten	Soup
Soup without legumes	Soep zonder peulvruchten	Soup
Butter / margarine (for bread)	Roomboter/margarine (brood)	Spreads
Low-fat margarine (for bread)	Halvarine (brood)	Spreads
Other spreads (for bread)	Overige soorten smeersel (brood)	Spreads
Chocolate spreads / sprinkles (for bread)	Chocoladebeleg	Sugar/Sweets
Other types of sweet spread / sprinkles (for bread)	Overige soorten zoet beleg	Sugar/Sweets
Sugar / syrup (for yoghurt)	Suiker/siroop in yoghurt ed.	Sugar/Sweets
Sugar (for coffee)	Suiker in koffie	Sugar/Sweets
Sugar / honey (for tea)	Suiker/honing in thee	Sugar/Sweets
Candy bars	Candybars	Sugar/Sweets
Chocolate	Chocolade	Sugar/Sweets
Candy	Snoep	Sugar/Sweets
Tea	Thee	Tea
Cooked vegetables without butter	Gekookte groente zonder boter	Vegetables
Stir-fried vegetables	Gewokte groente	Vegetables
Cooked vegetables with butter	Gekookte groente met boter	Vegetables
