# Supplementary material for: Habitual dietary intake of IBD patients differs from population controls: a case–control study
Source: Eur J Nutr. 2020 Apr 24;60(1):345–56. doi: 10.1007/s00394-020-02250-z (PMC7867519; doi:10.1007/s00394-020-02250-z)
Supplement: Supplementary file 2 — Supplementary file2 Table S2. Total protein intake of patients and controls (PDF 31 kb) [file 394_2020_2250_MOESM2_ESM.pdf]

Table S2. Total protein intake of patients and controls

	Disease Phenotype		Disease Activity		Controls
**Protein intake (g/kg)**	**UC**	**CD**	**Remission**	**Active Disease**	
Unknown intake*	2 (1.0)	2 (0.7)	3 (1.2)	1 (0.4)	0 (0)
<0.8	82 (39.6)	111 (38.8)	97 (38.6)	96 (39.8)	373 (28.9)
0.8-1.2	93 (44.9)	129 (45.1)	108 (43.0)	113 (46.9)	675 (52.3)
>1.2	30 (14.5)	44 (15.4)	43 (17.1)	31 (12.9)	243 (18.8)

Data is shown as frequency (%).

*Protein intake (g/kg) could not be calculated because of missing data on weight (kg).
