# Supplementary material for: Habitual dietary intake of IBD patients differs from population controls: a case–control study
Source: Eur J Nutr. 2020 Apr 24;60(1):345–56. doi: 10.1007/s00394-020-02250-z (PMC7867519; doi:10.1007/s00394-020-02250-z)
Supplement: Supplementary file 3 — Supplementary file3 Table S3. Multivariate analysis of food group intake in patients and controls (PDF 42 kb) [file 394_2020_2250_MOESM3_ESM.pdf]

**Table S3.** Multivariate analysis of food group intake in patients and controls

| | Disease Phenotype | | | | | | | | Disease Activity | | | | | | | |
|---|---|---|---|---|---|---|---|---|---|---|---|---|---|---|---|---|
| | Ulcerative colitis | | | | Crohn's disease | | | | Active disease | | | | Remission | | | |
| **Food Groups** | Exp (B) | CI | | Sign. | Exp (B) | CI | | Sign. | Exp (B) | CI | | Sign. | Exp (B) | CI | | Sign. |
| Alcohol[1-DP,DA] | 0.898 | 0.866 | 0.931 | 0.000* | 0.893 | 0.864 | 0.923 | 0.000* | 0.911 | 0.882 | 0.941 | 0.000* | 0.883 | 0.851 | 0.916 | 0.000* |
| Breads | 0.995 | 0.991 | 0.999 | 0.022* | 0.997 | 0.994 | 1.000 | 0.091 | 0.998 | 0.995 | 1.001 | 0.254 | 0.994 | 0.990 | 0.998 | 0.005* |
| Cereals | 1.012 | 0.999 | 1.025 | 0.069 | 0.989 | 0.974 | 1.004 | 0.151 | 0.999 | 0.986 | 1.013 | 0.933 | 0.999 | 0.985 | 1.014 | 0.930 |
| Cheese | 1.005 | 0.998 | 1.011 | 0.176 | 1.000 | 0.993 | 1.007 | 0.998 | 1.002 | 0.995 | 1.008 | 0.593 | 1.002 | 0.995 | 1.009 | 0.555 |
| Coffee | 0.998 | 0.997 | 0.999 | 0.000* | 1.000 | 0.999 | 1.001 | 0.919 | 0.999 | 0.998 | 1.000 | 0.005* | 1.000 | 0.999 | 1.000 | 0.323 |
| Dairy[1-DP] | 0.943 | 0.913 | 0.973 | 0.000* | 0.920 | 0.894 | 0.945 | 0.000* | 0.998 | 0.997 | 0.999 | 0.000* | 0.998 | 0.997 | 0.999 | 0.000* |
| Eggs | 0.998 | 0.986 | 1.011 | 0.807 | 1.004 | 0.994 | 1.015 | 0.407 | 0.999 | 0.987 | 1.011 | 0.915 | 1.009 | 0.998 | 1.021 | 0.094 |
| Fish | 1.002 | 0.990 | 1.014 | 0.750 | 1.006 | 0.996 | 1.016 | 0.228 | 0.998 | 0.987 | 1.009 | 0.659 | 1.008 | 0.997 | 1.018 | 0.166 |
| Fruits | 0.999 | 0.998 | 1.001 | 0.355 | 1.000 | 0.999 | 1.001 | 0.561 | 1.000 | 0.999 | 1.001 | 0.692 | 1.000 | 0.999 | 1.001 | 0.958 |
| Legumes | 1.005 | 0.995 | 1.014 | 0.345 | 1.005 | 0.997 | 1.014 | 0.234 | 1.003 | 0.994 | 1.012 | 0.539 | 1.005 | 0.996 | 1.015 | 0.258 |
| Meat | 1.005 | 1.001 | 1.010 | 0.019* | 1.001 | 0.997 | 1.005 | 0.582 | 1.005 | 1.001 | 1.009 | 0.020* | 1.001 | 0.997 | 1.006 | 0.630 |
| Nonalcoholic drinks | 1.000 | 0.999 | 1.001 | 0.473 | 1.001 | 1.000 | 1.001 | 0.037* | 1.000 | 1.000 | 1.001 | 0.367 | 1.000 | 1.000 | 1.001 | 0.323 |
| Nuts | 0.995 | 0.982 | 1.007 | 0.408 | 0.987 | 0.975 | 0.999 | 0.040* | 1.001 | 0.991 | 1.011 | 0.836 | 0.972 | 0.957 | 0.988 | 0.001* |
| Pasta | 0.993 | 0.984 | 1.003 | 0.157 | 0.986 | 0.977 | 0.995 | 0.002* | 0.995 | 0.986 | 1.003 | 0.213 | 0.981 | 0.971 | 0.992 | 0.000* |
| Pastry | 1.001 | 0.994 | 1.008 | 0.743 | 0.994 | 0.987 | 1.001 | 0.069 | 0.994 | 0.987 | 1.001 | 0.103 | 0.999 | 0.992 | 1.006 | 0.848 |
| Potatoes | 1.002 | 0.999 | 1.005 | 0.267 | 1.004 | 1.001 | 1.007 | 0.021* | 1.001 | 0.998 | 1.004 | 0.529 | 1.005 | 1.002 | 1.008 | 0.004* |
| Prepared meals[1-DA] | 0.999 | 0.995 | 1.002 | 0.426 | 0.993 | 0.990 | 0.997 | 0.001* | 0.793 | 0.731 | 0.861 | 0.000* | 0.831 | 0.762 | 0.906 | 0.000* |
| Rice | 0.993 | 0.986 | 1.000 | 0.064 | 0.995 | 0.989 | 1.002 | 0.138 | 0.992 | 0.986 | 0.999 | 0.028* | 0.997 | 0.990 | 1.004 | 0.367 |
| Sauces | 1.004 | 0.991 | 1.017 | 0.592 | 0.992 | 0.980 | 1.004 | 0.201 | 0.999 | 0.987 | 1.011 | 0.822 | 0.998 | 0.985 | 1.011 | 0.711 |
| Savory snacks | 1.003 | 0.992 | 1.014 | 0.600 | 1.010 | 1.000 | 1.019 | 0.042* | 1.008 | 0.998 | 1.017 | 0.119 | 1.007 | 0.997 | 1.017 | 0.165 |
| Soup | 1.002 | 0.999 | 1.005 | 0.200 | 1.001 | 0.999 | 1.004 | 0.340 | 1.003 | 1.000 | 1.005 | 0.033* | 1.000 | 0.997 | 1.003 | 0.932 |
| Spreads | 1.019 | 1.004 | 1.035 | 0.016* | 1.013 | 1.000 | 1.026 | 0.052 | 1.009 | 0.996 | 1.022 | 0.178 | 1.024 | 1.008 | 1.039 | 0.003* |
| Sugar& sweets | 1.006 | 1.000 | 1.012 | 0.069 | 1.008 | 1.002 | 1.014 | 0.005* | 1.009 | 1.003 | 1.015 | 0.002* | 1.006 | 1.000 | 1.012 | 0.063 |
| Tea | 1.000 | 0.999 | 1.001 | 0.782 | 1.000 | 0.999 | 1.001 | 0.906 | 1.000 | 0.999 | 1.001 | 0.687 | 1.000 | 0.999 | 1.001 | 0.946 |
| Vegetables | 1.000 | 0.997 | 1.003 | 0.879 | 0.999 | 0.997 | 1.002 | 0.615 | 0.999 | 0.997 | 1.002 | 0.654 | 1.000 | 0.997 | 1.003 | 0.974 |

Multinomial Logistic Regression Analysis is interpreted.

Model: gender, age, BMI, all 25 food groups.

[1] Observed variable is shown. Statistics were performed on √-transformed variable.

[DP] Variable was transformed when analyzing Disease Phenotype

[DA] Variable was transformed when analyzing Disease Activity

* Significant difference between subgroup vs controls ($p<0.05$)
